# The effect on congenital heart diseases of maternal *EPHX1* polymorphisms modified by polycyclic aromatic hydrocarbons exposure

**DOI:** 10.1097/MD.0000000000016556

**Published:** 2019-07-26

**Authors:** Jing Tao, Nana Li, Zhen Liu, Ying Deng, Xiaohong Li, Ming Chen, Jing Yu, Jun Zhu, Ping Yu, Yanping Wang

**Affiliations:** a National Center for Birth Defect Monitoring, West China Second University Hospital, Sichuan University; b Key Laboratory of Birth Defects and Related Diseases of Women and Children (Sichuan University), Ministry of Education, Chengdu, Sichuan; c Department of Ultrasound, Harbin Red Cross Central Hospital, Harbin, Heilongjiang; d Department of Pediatrics, Mianyang Central Hospital, Mianyang, Sichuan, China.

**Keywords:** congenital heart diseases, polycyclic aromatic hydrocarbons, *EPHX1*, polymorphism

## Abstract

Polycyclic aromatic hydrocarbons (PAHs) may be 1 of etiologic factors responsible for congenital heart diseases (CHDs). Variations of the microsomal epoxide hydrolase (*EPHX1*) gene, as well as their possible interactions with PAHs exposure, may increase susceptibility to CHDs.

This case–control study investigated the risk of CHDs in relation to the *EPHX1* polymorphisms and assessed the interactions between these polymorphisms and PAHs exposure in 357 mothers of CHDs fetuses and 270 control mothers. Logistic regression models for the risk of CHDs were applied to determine the effect of genetic polymorphisms using additive, recessive, and dominant genetic models, as well as gene-exposure interactions. Multiple testing was adjusted by applying the false discovery rate (FDR).

None of the maternal genetic polymorphisms of *EPHX1* was associated with CHDs occurrence. Only the single nucleotide polymorphism rs1051740 was associated with an increased risk of right-sided obstructive malformations under the recessive model (adjusted odds ratio [aOR] = 1.852, 95% confidence interval [CI]: 1.065, 3.22) before FDR correction. A possible modifying effect of PAHs exposure on genetic polymorphisms of *EPHX1* was found in susceptibility to CHDs, though no multiplicative-scale interactions between maternal exposure to PAHs and polymorphisms of *EPHX1* gene were seento affect the risk of CHDs.

The role of *EPHX1* gene polymorphisms for CHDs need to be further evaluated, in particularly by interacting with PAHs exposure.

## Abstract 2

Supplemental Digital Content is available in the text

## 1 Introduction

Congenital heart diseases (CHDs) are among the most common birth defects and the leading cause of birth defect-related mortality.^[1]^ Incidence of CHDs varies from approximately 8/1000 to 12/1000 live births.^[2,3]^ The etiology of CHDs, which have a vast range of cardiac defect phenotypes (e.g., septal defects, conotruncal heart defects), is thought to be determined by both environmental and genetic factors.^[4]^

Increasing evidence from epidemiological studies indicates that environmental pollutants (e.g., chemicals, air pollution) play an important role in the etiology of CHDs.^[5,6]^ Polycyclic aromatic hydrocarbons (PAHs) are ubiquitous environmental contaminants derived mostly from the incomplete combustion of coal, tobacco, diesel/gasoline-powered vehicle emissions, and other organic substances.^[7,8]^ In view of lower detoxification capacity of the fetus during early development, maternal exposure to PAHs may increase the risk of CHDs. Animal studies suggest prenatal exposure to PAHs can induce CHDs.^[9,10]^ In humans, several epidemiological studies have investigated the impact of maternal PAHs exposure on the risk of CHDs, but the results have been inconsistent. A large case-control study indicated no statistically significant association between potential maternal exposure to PAHs and CHDs among offspring.^[11]^ Two studies reported that PAH-containing particulate (PM2.5 and PM10) exposure during pregnancy was associated with a subgroup of CHDs (e.g., atrial septal defects, pulmonary valve stenosis).^[12,13]^ We also previously reported that PAHs exposure may increase the risk of CHDs.^[14]^

Accumulating evidence suggests that susceptibility gene displays a significant interaction with environmental pollutants in regard to disease risk.^[15–18]^ Thus, we hypothesized that maternal polymorphisms in genes encoding enzymes involved in the metabolism of PAHs may modulate fetal susceptibility to CHDs. The microsomal epoxide hydrolase (EPHX1) encoded by the *EPHX1* gene is an important enzyme. It is widely accepted that EPHX1 play a dual role in both the activation and the detoxification of PAHs and aromatic amines.^[19]^ Variations of *EPHX1* gene may alter enzymatic function,^[20]^ leading to several diseases such as cancers and birth defects.^[21–23]^ Such traits were mainly manifested in the presence of exogenous substance (such as tobacco smoke by-products) that cross the placenta,^[24]^ but there is little evidence regarding whether maternal *EPHX1* gene polymorphisms interact with PAHs exposure in susceptibility to CHDs.

We, therefore, conducted a hospital-based case–control study in China, with the primary objectives of further analysing the role of maternal *EPHX1* polymorphisms, as well as their possible interactions with PAHs exposure on modulating the risk of CHDs.

## 2 Materials and methods

### 2.1 Study population and epidemiological data collection

Subject recruitment was restricted to pregnant women from 6 tertiary maternal and child health hospitals in China. Cases were restricted to pregnant women having fetuses diagnosed with CHDs and without any extracardiac abnormalities determined by echocardiography and having a gestational age greater than 12 weeks. CHDs were further confirmed by humanitarian examination of the pathological anatomy for aborted fetuses, or by ultrasound examination performed within 30 postnatal days for born fetuses. Pregnancies resulting in multiple births and fetuses with syndromic diseases or chromosomal aberrations were excluded. CHDs cases were divided into 6 subgroups based on the anatomic lesions, including septal defects, conotruncal heart defects, right-sided obstructive malformations, left-sided obstructive malformations, anomalous pulmonary venous return, and other heart abnormalities.^[25]^ Controls were pregnant women having fetuses with no major congenital malformations diagnosed by echocardiography in the same hospital and having a gestational age greater than 12 weeks. A telephone follow-up was performed in all born cases and controls within 60 days.

From February 2010 to July 2015, 357 cases and 270 controls were recruited for this study. The flowchart of case and control inclusion and exclusion is shown in a prior study.^[14]^ All cases and controls were the same as those included in the prior study. All participants signed an informed consent. This research was approved by the Ethics Committee of Sichuan University (No. 2010004) and followed the tenets of the Declaration of Helsinki.

Maternal interviews were conducted during the antenatal examination by a trained interviewer using a standard questionnaire. As previously described,^[14]^ factors that could confound the relationship between PAHs exposure and CHDs were selected a priori from a set of characteristics. Potential confounders included maternal age at the time of the last menstrual period (years), gestational week (weeks), home or workplace renovation (yes or no), exposure to a factory or landfill nearby (<1000 meters, yes or no), cooking at home (often: ≥4 times/week, never, or occasional: 1∼4 times/week), parental smoking or environmental tobacco smoke (ETS) exposure (yes or no), maternal alcohol consumption (often: ≥1 time(s)/week, occasional: <1 time/week, or never), and use of folic acid supplements (yes or no). Ten milliliters of urine were collected from each participant in the morning and stored at −70°C until analysis. Four milliliters of blood were collected in EDTA from each participant by venepuncture and stored at −70°C until genotyping.

Urinary 1-hydroxypyrene-glucuronide (1-OHPG) is a sensitive exposure biomarker for low-level PAHs exposure and a suitable internal dose biomarker.^[26]^ We calculated the PAHs exposure status of pregnant women by measuring the concentration of 1-OHPG in urine. Before data analysis,^[14]^ we established the optimal cutoff values to maximize sensitivity and specificity using receiver operating characteristic curves: the cut-off value between subjects with high PAHs exposure and those with low PAHs exposure was 0.03186 μg/g Cr (specificity = 22.96%, sensitivity = 87.96%).

### 2.2 Genetic analysis

Genomic DNA was extracted from peripheral blood leukocytes using a QIAampDNA Blood Mini Kit (Qiagen, Cat. No. 51106, Germany). Single nucleotide polymorphisms (SNPs) in *EPHX1* gene were selected based on the following principal criteria:

(1) SNPs that have been reported to be significantly associated with various diseases or metabolic products of PAHs in human body in previous studies,^[23,24,27,28]^ and
(2) a minor allele frequency (MAF) >0.05 in Han Chinese.

Using the option of aggressive tagging with an r^2^ threshold of 0.8, 5 SNPs were selected for our study. The SNPs were genotyped using an improved multiplex ligation detection reaction (iMLDR) technique that was newly developed by Genesky Biotechnologies Inc. (Shanghai, China). Genotyping was successfully performed on all study subjects. For quality-control assessment, genotyping was repeated in 10% of samples, and the consistency rate was 100%. More detailed information about the studied genetic variants and genotyping is presented in supplementary materials (see Table S1, Supplementary Content, which displays the information of SNPs in *EPHX1* gene)

### 2.3 Statistical analysis

Chi-square statistics or Mann–Whitney test of nonparametric test was used to evaluate differences in covariates between the cases and controls. Deviations from Hardy–Weinberg equilibrium (HWE) in the controls were assessed using Plink software (http://pngu.mgh.harvard.edu/∼purcell/plink/). The pairwise linkage disequilibrium (LD) patterns of *EPHX1* gene were estimated using Haploview 4.2 software. Logistic regression analysis performed by Plink software was performed to examine the effect of genetic polymorphisms on the risk of CHDs and, in additive, recessive and dominant models. The effects of the gene-exposure interactions on occurrence of CHDs were evaluated by logistic models using SPSS version 16.0 software (SPSS Inc., IBM, Chicago, IL). Two-sided *P* <.05 was considered statistically significant. To robustly correct for multiple hypothesis tests, we considered the widely used Benjamini–Hochberg (BH) procedure to control the false discovery rate (FDR),^[29]^ and calculated the FDR-adjusted *P* values at a level of .05.^[30]^

## 3 Results

### 3.1 Characteristics of study participants

Six hundred twenty-seven participants (357 CHD cases and 270 controls) were included in this analysis. Among CHD cases, there were 235 cases (65.83%) with septal defects, 160 cases (44.82%) with conotruncal heart defects, 114 cases (31.93%) with right-sided obstructive malformations, 72 cases (20.17%) with left-sided obstructive malformations, 64 cases (17.93%) with anomalous pulmonary venous return, and 102 cases (28.57%) with other cardiac structural abnormalities.

Table 1 displays the frequency distribution of baseline characteristics of the participants. There were significant differences between the 2 groups with respect to gestational week, cooking at home, maternal alcohol consumption, folic acid supplementation, and urinary 1-OHPG levels.

**Table 1:**
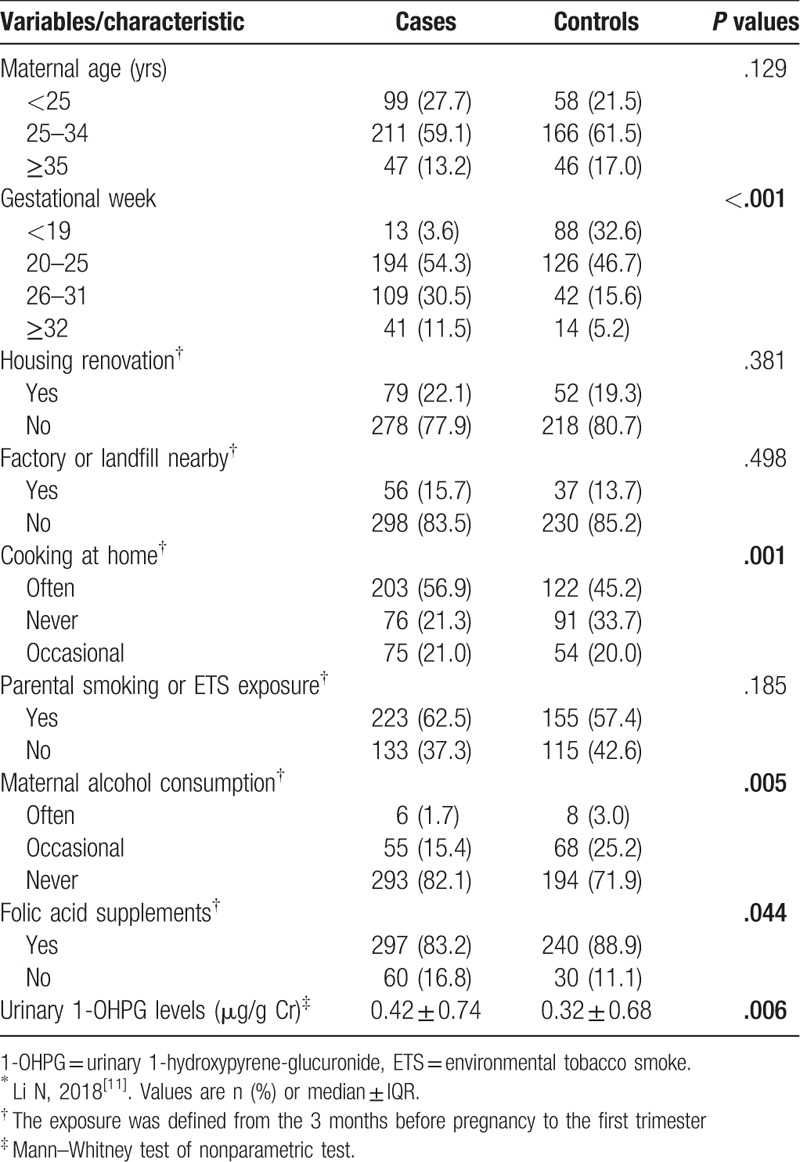
Characteristics of the case and control participants^∗^.

### 3.2 Association between maternal *EPHX1* polymorphisms and risk of CHDs

Each of the 5 maternal polymorphisms of *EPHX1* demonstrated conformance to HWE in our controls (*P* >.05) (see Table S2, Supplementary Content, which shows HWE evaluation of SNPs in *EPHX1* gene), indicating good representativeness of our study population. Linkage disequilibrium parameters (r^2^) for 6 combinations of the 5 SNPs were 0, therefore, 6 combinations satisfied the linkage equilibrium (see Figure S1, Supplementary Content, which displays the results of linkage disequilibrium parameters analysis).

No significant association was observed between any genetic polymorphism of *EPHX1* and the risk of CHDs (Table 2) and any specific type (Table S3, Supplementary Content, which illustrates the risk of each CHD type associated with any genetic polymorphism of *EPHX1*) after FDR correction. Interestingly, the SNP rs1051740 was associated with an increased risk of right-sided obstructive malformations under the recessive model (adjusted odds ratio [aOR] = 1.852, 95% confidence interval [CI]: 1.065, 3.22) before FDR correction, but the association disappeared after FDR correction (see Table S3-3, Supplementary Content, which illustrates the risk of right-sided obstructive malformations associated with any genetic polymorphism of *EPHX1*).

**Table 2:**
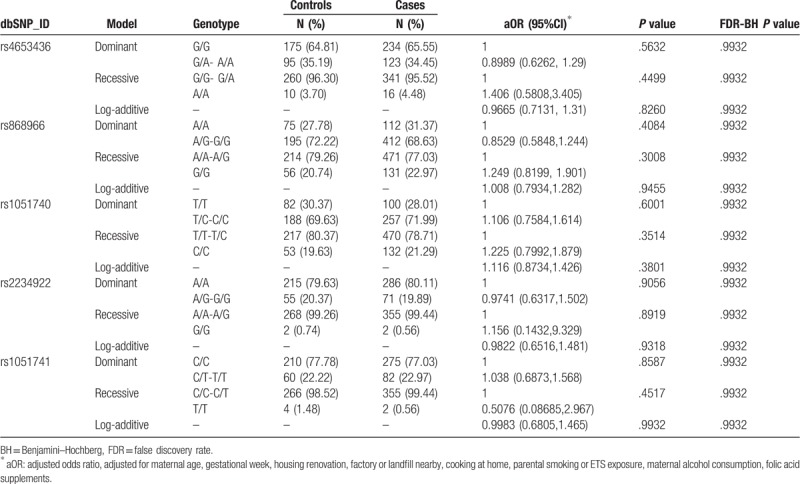
Association between maternal genotypes and risk of congenital heart diseases.

### 3.3 Interaction between maternal *EPHX1* polymorphisms and PAHs exposure on the risk of CHDs

Assuming a dominant genetic model (minor allele considered the risk allele) and a 1 df association test, the results of interaction analyses between maternal *EPHX1* polymorphisms and exposure to PAHs on the risk of CHDs are shown in Table 3. We found that a combination of high PAHs exposure and the *EPHX1* rs4653436 with G/G genotype increased the odds of CHDs (aOR, 1.990; 95% CI, 1.107, 3.578). And a combination of high PAHs exposure and the T/T, and the T/C or C/C genotypes of SNP rs1051740 in the *EPHX1* gene seemed to have a high influence on the risk of CHDs with adjusted ORs of 3.642 (1.517, 8.744) and 3.606 (1.578, 8.239), respectively. Moreover, a combination of high PAHs exposure and the A/A, and the A/G or G/G genotypes of SNP rs2234922 in the *EPHX1* gene found to have a modest influence on the risk of CHDs with adjusted ORs of 2.252 (1.342, 3.781) and 2.046 (1.086, 3.856), respectively. Similar patterns of effect for high PAHs exposure and the C/C and the C/T or T/T genotypes of SNP rs1051741 in the *EPHX1* gene were observed for CHDs with adjusted ORs of 1.86 (1.094, 3.165) and 2.105 (1.118, 3.966), respectively. However, no multiplicative-scale interactions were observed between maternal exposure to PAHs and *EPHX1* polymorphisms on the risk of CHDs before or after FDR correction.

**Table 3:**
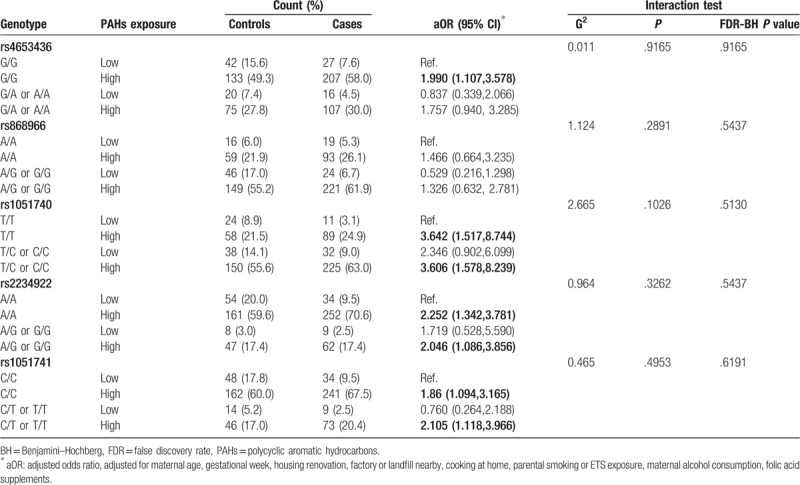
Interaction between maternal polycyclic aromatic hydrocarbons exposure and genotypes on the risk of congenital heart diseases.

## 4 Discussion

In this case-control study, we fail to detect the association between any genetic polymorphism of *EPHX1* and the risk of CHDs and specific subgroups. Only the SNP rs1051740 was found to be associated with an increased risk of right-sided obstructive malformations before FDR correction, while others were not. In addition, a possible modifying effect of PAHs exposure on genetic polymorphisms of *EPHX1* was found in susceptibility to CHDs, adding to the evidence that PAHs exposure and *EPHX1* polymorphisms acting together in risk of CHDs.

The SNP rs1051740 in the *EPHX1* gene consists of a T >C substitution in exon 3, leading to a change from tyrosine to histidine amino acid residue at position 113 (Tyr113His) of the epoxide hydrolase protein. Using in vitro expression, Hassett et al described that the 113His isoform has 39% reduced mEH activity.^[20]^ Thus, it is likely that the lower enzyme activity resulting from the *EPHX1* rs1051740 leads to cellular DNA damage and contributes to increased susceptibility to birth defects. One study reported an association between the maternal *EPHX1* rs1051740 polymorphism and the risk of craniofacial abnormalities in offspring.^[23]^ Another study in 2012 showed that the maternal *EPHX1* rs1051740 genotype was associated with increased risks for childhood medulloblastoma.^[27]^ In the present study, we found that pregnant women with the *EPHX1* rs1051740 polymorphism conferred the risk of right-sided obstructive malformations before FDR correction. Because CHDs have different histological types, further studies with larger case sample are needed to give more convincing results on behalf of identifying the association between the *EPHX1* rs1051740 polymorphism and risk of different CHD subtypes.

For all other genetic polymorphisms, no significant associations with CHDs were observed. For the *EPHX1* rs2234922, the lack of associations with other diseases was observed in previous studies. One study did not find the association of rs2234922 with lung cancer.^[28]^ Similarly, Lakhdar et al showed that there is no significant correlation between rs2234922 and chronic obstructive pulmonary disease.^[31]^

An interesting finding was an increased risk of CHDs associated with maternal *EPHX1* polymorphisms in individuals with high PAHs exposure. There have been a few reports on SNPs in the E*PHX1* gene, environmental exposure and the impacts on some diseases. One study found that children with the high-risk genotype for *EPHX1* rs2234922 genotype exposed to paternal smoking for >3 hours per day were 3.18 times as likely as unexposed children to develop a childhood brain tumor.^[24]^ Another study reported that smokers carrying *EPHX1* rs1051740 with Tyr/Tyr and Tyr/His genotype were at increased prostate cancer risk relative to non-smokers with the Tyr/Tyr genotype.^[19]^ Based on the data and our study findings, we hypothesized that xenobiotic substance might influence the role of maternal *EPHX1* gene, and increase metabolic intermediates (active oxygen species and DNA or protein binding adducts) in fetal tissues which in turn could affect fetal development. Further study with a larger sample is needed to clarify the interactions between *EPHX1* polymorphisms and PAHs exposure.

This study had several strengths. This study was the first to evaluate the effect of the interaction between maternal *EPHX1* polymorphisms and PAHs exposure on the risk of CHDs and CHD subtypes. Second, the use of urinary 1-hydroxypyrene-glucuronide (1-OHPG) as a biomarker to estimate PAHs exposure level was advantageous because it integrated all possible exposure routes, for example, inhalation, ingestion, and dermal contact, and can account for issues resulting from inter-individual differences in genetics and metabolism compared to external measures of exposure.^[32]^

Our study has several limitations. First, small sample size may limit statistical power to estimate the possible CHD risk with the SNPs of *EPHX1* gene and their interaction with PAHs exposure. Second, we cannot exclude the possibility that these findings may be confounded by other unmeasured toxin or risk factors, and by alternative or additional genes. Therefore, the results of this study need to be interpreted with caution.

## 5 Conclusions

Overall, our study found the *EPHX1* rs1051740 was associated with an increased risk of right-sided obstructive malformations. A possible modifying effect of PAHs exposure on genetic polymorphism of *EPHX1* was found in susceptibility to CHDs. Regarding the interplay with PAHs exposure, the role of *EPHX1* gene variation in CHDs requires further observational and mechanistic studies.

## Acknowledgments

We are indebted to the pediatric cardiologists, geneticists, and epidemiologists who collaborated in this program and made the study possible. We thank the obstetricians, pediatricians, pathologists, experimental technicians, and other participants involved in the project for recruiting the case and control participants and collecting the data. We thank all participating families for their cooperation and for providing personal information. We also thank the reviewers for their helpful comments.

## Author contributions

**Data curation:** Ming Chen, Jing Yu.

**Formal analysis:** Jing Tao, Nana Li.

**Methodology:** Zhen Liu, Ying Deng.

**Software:** Xiaohong Li, Jun Zhu.

**Writing – original draft:** Jing Tao.

**Writing – review & editing:** Ping Yu, Yanping Wang.

## Supplementary Material

Supplemental Digital Content
