# Supplementary material for: The effect on congenital heart diseases of maternal EPHX1 polymorphisms modified by polycyclic aromatic hydrocarbons exposure
Source: Medicine (Baltimore). 2019 Jul 26;98(30):e16556. doi: 10.1097/MD.0000000000016556 (PMC6709072; doi:10.1097/MD.0000000000016556)
Supplement: Supplemental Digital Content [file medi-98-e16556-s001.doc]

**The effect on congenital heart diseases of maternal *EPHX1* polymorphisms modified by polycyclic aromatic hydrocarbons exposure**

Jing Tao, MD^a,b*^,Nana Li, PhD^a,b *^, Zhen Liu, PhD^a,b^, Ying Deng, PhD^a,b^, Xiaohong Li, PhD^a,b^, Ming Chen, MD^c^, Jing Yu, MD^d^, Jun Zhu, MD^a,b^, Ping Yu, PhD^a,b^, Yanping Wang, MD^a,b^

^a^National Center for Birth Defect Monitoring, West China Second University Hospital, Sichuan University, Sec.3 No.17, South RenMin Road, Chengdu, Sichuan, China

^b^ Key Laboratory of Birth Defects and Related Diseases of Women and Children (Sichuan University), Ministry of Education, Chengdu, Sichuan, China.

^c^Department of Ultrasound, Harbin Red Cross Central Hospital, Harbin, Heilongjiang, China.

^d^Department of pediatrics, Mianyang Central Hospital, Mianyang, Sichuan, China.

**Table S1 The information of SNPs in *EPHX1* gene**

| **dbSNP_ID** | **Allele** | **chr** | **Chr. Position** | **Ref mRNA** | **SNP Property** | **Functional Change** | **HapMap-HCB** |
| --- | --- | --- | --- | --- | --- | --- | --- |
| rs4653436 | G/A | 1 | 225995211 | NM_000120.3 | 5'-flanking | / | 0.254 |
| rs868966 | A/G | 1 | 226006098 | NM_000120.3 | 5'-flanking | / | 0.438 |
| rs1051740 | T/C | 1 | 226019633 | NM_000120.3 | nonsynon_exon3 | p.Tyr113His | 0.427 |
| rs2234922 | A/G | 1 | 226026406 | NM_000120.3 | nonsynon_exon4 | p.His139Arg | 0.106 |
| rs1051741 | C/T | 1 | 226032229 | NM_000120.3 | synon_exon8 | p.Asn357Asn | 0.117 |

**Table S2 Hardy-Weinberg equilibrium evaluation of SNPs in *EPHX1* gene**

| dbSNP_ID | Group | Genotype count（frequency %） | | | HWE *P* |
| --- | --- | --- | --- | --- | --- |
| rs4653436 |  | G/G | G/A | A/A |  |
|  | Controls | 175（64.81） | 85（31.48） | 10（3.70） | 1 |
|  | Cases | 234（65.55） | 10729.97） | 16（4.48） |  |
| rs868966 |  | A/A | A/G | G/G |  |
|  | Controls | 75（27.78） | 139（51.48） | 56（20.74） | 0.6253 |
|  | Cases | 112（31.37） | 163（45.66） | 82（11.97） |  |
| rs1051740 |  | T/T | T/C | C/C |  |
|  | Controls | 82（30.37） | 135（50.00） | 53（19.63） | 0.9023 |
|  | Cases | 100（28.01） | 181（50.70） | 76（21.29） |  |
| rs2234922 |  | A/A | A/G | G/G |  |
|  | Controls | 215（79.63） | 53（19.63） | 2（0.74） | 0.7496 |
|  | Cases | 286（80.11） | 69（19.33） | 2（0.56） |  |
| rs1051741 |  | C/C | C/T | T/T |  |
|  | Controls | 210（77.78） | 56（20.74） | 4（1.48） | 0.7757 |
|  | Cases | 275（77.03） | 80（22.41） | 2（0.56） |  |

**Table S3-1** Association between maternal genotypes and risk of septal defects

| dbSNP ID | Model | Genotype | Controls | **septal defects** | aOR(95%CI) ^a^ | *P*-value | FDR-BH *P* value |
| --- | --- | --- | --- | --- | --- | --- | --- |
|  |  |  | N(%) | N(%) |  |  |  |
| rs4653436 | Dominant | G/G | 175（64.81） | 150（63.83） | 1 | .8671 | .9901 |
|  |  | G/A- A/A | 95（35.19） | 85（36.17） | 0.9668(0.6506,1.436) |  |  |
|  | Recessive | G/G- G/A | 260（96.30） | 225（95.74） | 1 | .6172 | .9901 |
|  |  | A/A | 10（3.70） | 10（4.26） | 1.28(0.4866,3.364) |  |  |
|  | Log-additive | - | - | - | 1.005(0.7188,1.406) | .9748 | .9901 |
| rs868966 | Dominant | A/A | 75（27.78） | 78（33.19） | 1 | .2881 | .9901 |
|  |  | A/G-G/G | 195（72.22） | 157（66.81） | 0.7992(0.5284,1.209) |  |  |
|  | Recessive | A/A-A/G | 214（79.26） | 187（79.57） | 1 | .7438 | .9901 |
|  |  | G/G | 56（20.74） | 48（20.43） | 1.082(0.6743,1.736) |  |  |
|  | Log-additive | - | - | - | 0.9329(0.7127,1.221) | .6134 | .9901 |
| rs1051740 | Dominant | T/T | 82（30.37） | 61（25.96） | 1 | .4190 | .9901 |
|  |  | T/C-C/C | 188（69.63） | 174（74.04） | 1.19(0.7801,1.816) |  |  |
|  | Recessive | T/T-T/C | 217（80.37） | 182（77.45） | 1 | .2538 | .9901 |
|  |  | C/C | 53（19.63） | 53（22.55） | 1.313(0.8226,2.095) |  |  |
|  | Log-additive | - | - | - | 1.179(0.8979,1.549) | .2358 | .9901 |
| rs2234922 | Dominant | A/A | 215（79.63） | 190（80.85） | 1 | .7410 | .9901 |
|  |  | A/G-G/G | 55（20.37） | 45（19.15） | 0.9221(0.5701,1.491) |  |  |
|  | Recessive | A/A-A/G | 268（99.26） | 234（99.57） | 1 | .9901 | .9901 |
|  |  | G/G | 2（0.74） | 1（0.43） | 0.9842(0.08025,12.07) |  |  |
|  | Log-additive | - | - | - | 0.9282(0.5862,1.47) | .7507 | .9901 |
| rs1051741 | Dominant | C/C | 210（77.78） | 180（76.60） | 1 | .7571 | .9901 |
|  |  | C/T-T/T | 60（22.22） | 55（23.40） | 1.074(0.6839,1.686) |  |  |
|  | Recessive | C/C-C/T | 266（98.52） | 234（99.57） | 1 | .4386 | .9901 |
|  |  | T/T | 4（1.48） | 1（0.43） | 0.412(0.04369,3.885) |  |  |
|  | Log-additive | - | - | - | 1.024(0.6722,1.56) | .9117 | .9901 |

^a^ aOR: adjusted odds ration, adjusted for maternal age, gestational week, housing renovation, factory or landfill nearby, cooking at home, parental smoking or ETS exposure, maternal alcohol consumption, folic acid supplements.

**Table S3-2 Association between maternal genotypes and risk of** conotruncal heart defects

| dbSNP_ID | Model | Genotype | Controls | **conotruncal heart defects** | aOR(95%CI) ^a^ | *P*-value | FDR-BH *P* value |
| --- | --- | --- | --- | --- | --- | --- | --- |
|  |  |  | N(%) | N(%) |  |  |  |
| rs4653436 | Dominant | G/G | 175（64.81） | 103（64.38） | 1 | .9271 | .9969 |
|  |  | G/A- A/A | 95（35.19） | 57（35.62） | 0.9792(0.6244,1.536) |  |  |
|  | Recessive | G/G- G/A | 260（96.30） | 151（94.38） | 1 | .4046 | .9969 |
|  |  | A/A | 10（3.70） | 9（5.62） | 1.547(0.5546,4.313) |  |  |
|  | Log-additive | - | - | - | 1.044(0.7178,1.518) | .8219 | .9969 |
| rs868966 | Dominant | A/A | 75（27.78） | 49（30.63） | 1 | .3802 | .9969 |
|  |  | A/G-G/G | 195（72.22） | 111（69.37） | 0.8082(0.5024,1.3) |  |  |
|  | Recessive | A/A-A/G | 214（79.26） | 127（79.38） | 1 | .5033 | .9969 |
|  |  | G/G | 56（20.74） | 33（20.62） | 1.202(0.7013,2.06) |  |  |
|  | Log-additive | - | - | - | 0.9706(0.7113,1.324) | .8509 | .9969 |
| rs1051740 | Dominant | T/T | 82（30.37） | 51（31.88） | 1 | .5431 | .9969 |
|  |  | T/C-C/C | 188（69.63） | 109（68.12） | 0.8664(0.5457,1.376) |  |  |
|  | Recessive | T/T-T/C | 217（80.37） | 125（78.13） | 1 | .4109 | .9969 |
|  |  | C/C | 53（19.63） | 35（21.87） | 1.251(0.7337,2.132) |  |  |
|  | Log-additive | - | - | - | 1.01(0.7455,1.369) | .9475 | .9969 |
| rs2234922 | Dominant | A/A | 215（79.63） | 132（82.50） | 1 | .6731 | .9969 |
|  |  | A/G-G/G | 55（20.37） | 28（17.50） | 0.887(0.5082,1.548) |  |  |
|  | Recessive | A/A-A/G | 268（99.26） | 159（99.38） | 1 | .9969 | .9969 |
|  |  | G/G | 2（0.74） | 1（0.62） | 1.005(0.07642,13.22) |  |  |
|  | Log-additive | - | - | - | 0.899(0.5314,1.521) | .6915 | .9969 |
| rs1051741 | Dominant | C/C | 210（77.78） | 127（79.38） | 1 | .7340 | .9969 |
|  |  | C/T-T/T | 60（22.22） | 33（20.62） | 0.9125(0.5382,1.547) |  |  |
|  | Recessive | C/C-C/T | 266（98.52） | 159（99.38） | 1 | .5751 | .9969 |
|  |  | T/T | 4（1.48） | 1（0.62） | 0.522(0.05377,5.068) |  |  |
|  | Log-additive | - | - | - | 0.8941(0.549,1.456) | .6528 | .9969 |

^a^ aOR: adjusted odds ration, adjusted for maternal age, gestational week, housing renovation, factory or landfill nearby, cooking at home, parental smoking or ETS exposure, maternal alcohol consumption, folic acid supplements.

**Table S3-3** Association between maternal genotypes and risk of right-sided obstructive malformations

| dbSNP_ID | Model | Genotype | Controls | **right-sided obstructive malformations** | | aOR(95%CI) ^a^ | *P*-value | FDR-BH *P* value |
| --- | --- | --- | --- | --- | --- | --- | --- | --- |
|  |  |  | N(%) | N(%) |  | |  |  |
| rs4653436 | Dominant | G/G | 175（64.81） | 72（63.16） | | 1 | .9216 | .9990 |
|  |  | G/A- A/A | 95（35.19） | 42（36.84） | | 1.025(0.6293,1.669) |  |  |
|  | Recessive | G/G- G/A | 260（96.30） | 109（95.61） | | 1 | .6880 | .9990 |
|  |  | A/A | 10（3.70） | 5（4.39） | | 1.274(0.3905,4.157) |  |  |
|  | Log-additive | - | - | - | | 1.048(0.6921,1.587) | .8248 | .9990 |
| rs868966 | Dominant | A/A | 75（27.78） | 37（32.46） | | 1 | .3787 | .9990 |
|  |  | A/G-G/G | 195（72.22） | 77（67.54） | | 0.7942(0.4755,1.327) |  |  |
|  | Recessive | A/A-A/G | 214（79.26） | 87（76.32） | | 1 | .1735 | .8675 |
|  |  | G/G | 56（20.74） | 27（23.68） | | 1.491(0.8388,2.65) |  |  |
|  | Log-additive | - | - | - | | 1.037(0.7389,1.454) | .8351 | .9990 |
| rs1051740 | Dominant | T/T | 82（30.37） | 29（25.44） | | 1 | .3034 | .9990 |
|  |  | T/C-C/C | 188（69.63） | 85（74.56） | | 1.321(0.7774,2.245) |  |  |
|  | Recessive | T/T-T/C | 217（80.37） | 83（72.81） | | 1 | .0292 | .3975 |
|  |  | C/C | 53（19.63） | 31（27.19） | | **1.852(1.065,3.22)** |  |  |
|  | Log-additive | - | - | - | | 1.395(0.9957,1.954) | .0530 | .3975 |
| rs2234922 | Dominant | A/A | 215（79.63） | 93（81.58） | | 1 | .7351 | .9990 |
|  |  | A/G-G/G | 55（20.37） | 21（18.42） | | 0.8997(0.4879,1.659) |  |  |
|  | Recessive | A/A-A/G | 268（99.26） | 114（100.00） | | 1 | .9990 | .9990 |
|  |  | G/G | 2（0.74） | 0（0.00） | | - |  |  |
|  | Log-additive | - | - | - | | 0.8665(0.4785,1.569) | .6363 | .9990 |
| rs1051741 | Dominant | C/C | 210（77.78） | 89（78.07） | | 1 | .8504 | .9990 |
|  |  | C/T-T/T | 60（22.22） | 25（21.93） | | 0.9457(0.5297,1.689) |  |  |
|  | Recessive | C/C-C/T | 266（98.52） | 114（100.00） | | 1 | .9986 | .9990 |
|  |  | T/T | 4（1.48） | 0（0.00） | | - |  |  |
|  | Log-additive | - | - | - | | 0.8848(0.5121,1.529) | .6611 | .9605 |

^a^ aOR: adjusted odds ration, adjusted for maternal age, gestational week, housing renovation, factory or landfill nearby, cooking at home, parental smoking or ETS exposure, maternal alcohol consumption, folic acid supplements.

**Table S3-4** Association between maternal genotypes and risk of left-sided obstructive malformations

| dbSNP_ID | Model | Genotype | Controls | **left-sided obstructive malformations** | | aOR(95%CI) ^a^ | *P*-value | FDR-BH *P* value |
| --- | --- | --- | --- | --- | --- | --- | --- | --- |
|  |  |  | N(%) | N(%) |  | |  |  |
| rs4653436 | Dominant | G/G | 175（64.81） | 47（65.28） | | 1 | .6847 | .9991 |
|  |  | G/A- A/A | 95（35.19） | 25（34.72） | | 0.8826(0.4831,1.613) |  |  |
|  | Recessive | G/G- G/A | 260（96.30） | 70（97.22） | | 1 | .8563 | .9991 |
|  |  | A/A | 10（3.70） | 2（2.78） | | 0.8603(0.1687,4.386) |  |  |
|  | Log-additive | - | - | - | | 0.8959(0.5299,1.515) | .6815 | .9991 |
| rs868966 | Dominant | A/A | 75（27.78） | 16（22.22） | | 1 | .4091 | .9991 |
|  |  | A/G-G/G | 195（72.22） | 56（77.78） | | 1.329(0.6765,2.611) |  |  |
|  | Recessive | A/A-A/G | 214（79.26） | 56（77.78） | | 1 | .2341 | .8779 |
|  |  | G/G | 56（20.74） | 16（22.22） | | 1.525(0.7608,3.058) |  |  |
|  | Log-additive | - | - | - | | 1.307(0.8535,2.002) | .2180 | .8779 |
| rs1051740 | Dominant | T/T | 82（30.37） | 18（25.00） | | 1 | .4311 | .9991 |
|  |  | T/C-C/C | 188（69.63） | 54（75.00） | | 1.3(0.6768,2.496) |  |  |
|  | Recessive | T/T-T/C | 217（80.37） | 54（75.00） | | 1 | .2297 | .8779 |
|  |  | C/C | 53（19.63） | 18（25.00） | | 1.526(0.7655,3.044) |  |  |
|  | Log-additive | - | - | - | | 1.295(0.8523,1.967) | .2259 | .8779 |
| rs2234922 | Dominant | A/A | 215（79.63） | 57（79.17） | | 1 | .8617 | .9991 |
|  |  | A/G-G/G | 55（20.37） | 15（20.83） | | 1.065(0.5247,2.161) |  |  |
|  | Recessive | A/A-A/G | 268（99.26） | 72（100.00） | | 1 | .9991 | .9991 |
|  |  | G/G | 2（0.74） | 0（0.00） | | - |  |  |
|  | Log-additive | - | - | - | | 1.025(0.5165,2.035) | .9435 | .9991 |
| rs1051741 | Dominant | C/C | 210（77.78） | 54（75.00） | | 1 | .7358 | .9991 |
|  |  | C/T-T/T | 60（22.22） | 18（25.00） | | 1.123(0.5723,2.204) |  |  |
|  | Recessive | C/C-C/T | 266（98.52） | 72（100.00） | | 1 | .9987 | .9991 |
|  |  | T/T | 4（1.48） | 0（0.00） | | - |  |  |
|  | Log-additive | - | - | - | | 1.041(0.5528,1.96) | .9013 | .9991 |

^a^ aOR: adjusted odds ration, adjusted for maternal age, gestational week, housing renovation, factory or landfill nearby, cooking at home, parental smoking or ETS exposure, maternal alcohol consumption, folic acid supplements.

**Table S3-5** Association between maternal genotypes and risk of anomalous pulmonary venous return

| dbSNP_ID | Model | Genotype | Controls | **anomalous pulmonary venous return** | | aOR(95%CI) ^a^ | *P*-value | FDR-BH *P* value |
| --- | --- | --- | --- | --- | --- | --- | --- | --- |
|  |  |  | N(%) | N(%) |  | |  |  |
| rs4653436 | Dominant | G/G | 175（64.81） | 40（62.50） | | 1 | .7733 | .9991 |
|  |  | G/A- A/A | 95（35.19） | 24（37.50） | | 0.9104(0.4806,1.725) |  |  |
|  | Recessive | G/G- G/A | 260（96.30） | 60（93.75） | | 1 | .2780 | .9991 |
|  |  | A/A | 10（3.70） | 4（6.25） | | 2.08(0.5539,7.811) |  |  |
|  | Log-additive | - | - | - | | 1.042(0.6129,1.772) | .8787 | .9991 |
| rs868966 | Dominant | A/A | 75（27.78） | 22（34.38） | | 1 | .3057 | .9991 |
|  |  | A/G-G/G | 195（72.22） | 42（65.63） | | 0.7062(0.363,1.374) |  |  |
|  | Recessive | A/A-A/G | 214（79.26） | 54（84.38） | | 1 | .9765 | .9991 |
|  |  | G/G | 56（20.74） | 10（15.62） | | 1.013(0.4411,2.324) |  |  |
|  | Log-additive | - | - | - | | 0.8505(0.5335,1.356) | .4961 | .9991 |
| rs1051740 | Dominant | T/T | 82（30.37） | 18（28.13） | | 1 | .8055 | .9991 |
|  |  | T/C-C/C | 188（69.63） | 46（71.87） | | 1.089(0.5517,2.151) |  |  |
|  | Recessive | T/T-T/C | 217（80.37） | 48（75.00） | | 1 | .2120 | .9991 |
|  |  | C/C | 53（19.63） | 16（25.00） | | 1.603(0.7641,3.362) |  |  |
|  | Log-additive | - | - | - | | 1.219(0.7822,1.901) | .3812 | .9991 |
| rs2234922 | Dominant | A/A | 215（79.63） | 55（85.94） | | 1 | .6105 | .9991 |
|  |  | A/G-G/G | 55（20.37） | 95（14.06） | | 0.8039(0.347,1.862) |  |  |
|  | Recessive | A/A-A/G | 268（99.26） | 64（100.00） | | 1 | .9991 | .9991 |
|  |  | G/G | 2（0.74） | 0（0.00） | | - |  |  |
|  | Log-additive | - | - | - | | 0.7828(0.3462,1.77) | .5564 | .9991 |
| rs1051741 | Dominant | C/C | 210（77.78） | 51（79.69） | | 1 | .9545 | .9991 |
|  |  | C/T-T/T | 60（22.22） | 13（20.31） | | 0.9782(0.4591,2.085) |  |  |
|  | Recessive | C/C-C/T | 266（98.52） | 64（100.00） | | 1 | .9987 | .9991 |
|  |  | T/T | 4（1.48） | 0（0.00） | | - |  |  |
|  | Log-additive | - | - | - | | 0.9206(0.4509,1.88) | .8203 | .9991 |

^a^ aOR: adjusted odds ration, adjusted for maternal age, gestational week, housing renovation, factory or landfill nearby, cooking at home, parental smoking or ETS exposure, maternal alcohol consumption, folic acid supplements.

| 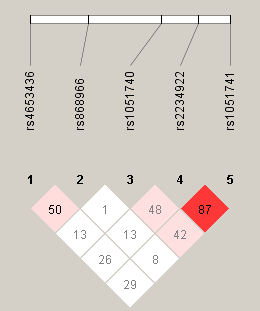 | 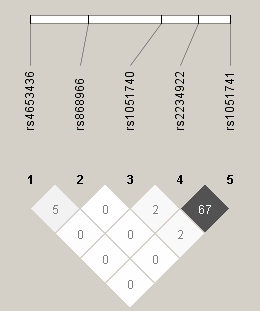 |
| --- | --- |
| EPHX1: D’ | R^2^ |

**Fig S1: Linkage Disequilibrium Analysis of SNPs**
